# Frequency and power dependence of ultrasonic degassing

**DOI:** 10.1016/j.ultsonch.2021.105890

**Published:** 2021-12-22

**Authors:** Yoshiyuki Asakura, Keiji Yasuda

**Affiliations:** a Honda Electronics Co., Ltd., Toyohashi, Aichi 441-3193, Japan; b Department of Chemical Systems Engineering, Graduate School of Engineering, Nagoya University, Nagoya, Aichi 464-8603, Japan

**Keywords:** Ultrasonic degassing, Ultrasonic power, Ultrasonic frequency, Quenching

## Abstract

We investigated the time variation of ultrasonic degassing for air-saturated water and degassed water with a sample volume of 100 mL at frequencies of 22, 43, 129, 209, 305, 400, 514, 1018, and 1960 kHz and ultrasonic power of 15 W. Ultrasonic degassing was evaluated by dissolved oxygen concentration. Ultrasonic degassing was also investigated at a frequency of 1018 kHz and ultrasonic powers of 5, 10, 15, and 20 W. The dissolved oxygen concentration varied with the ultrasonic irradiation time and became constant after prolonged ultrasonic irradiation. The constant dissolved oxygen concentration value depended on the frequency and ultrasonic power but not the initial dissolved oxygen concentration. The degassing rate at 101.3 kPa was higher in the frequency range of 200 kHz to 1 MHz. The frequency dependence of the degassing rate was almost the same as that of the sonochemical efficiency obtained by the potassium iodide (KI) method. Ultrasonic degassing in the frequency range of 22–1960 kHz was also investigated under reduced pressure of 5 kPa. Degassing was accelerated when ultrasonic irradiation was applied under reduced pressure. However, under a reduced pressure of 5 kPa, the lower the frequencies, the higher is the degassing rate. The sonochemical reaction rate was examined by the KI method for varying dissolved air concentrations before ultrasonic irradiation. Cavitation did not occur when the initial dissolved oxygen concentration was less than 2 mg·L^−1^. Therefore, the lower limit of ultrasonic degassing under 101.3 kPa equals 2 mg·L^−1^ dissolved oxygen concentration. A model equation for the time variation of dissolved oxygen concentration due to ultrasonic irradiation was developed, and the degassing mechanism was discussed.

## Highlights

• Ultrasonic degassing was investigated at 22–1960 kHz under 101.3 and 5 kPa.
• Ultrasonic degassing was evaluated by dissolved oxygen concentration (DO)
• Degassing increased in the frequency range of 200–1000 kHz under 101.3 kPa.
• The minimum value of DO by ultrasonic degassing at 101.3 kPa was 2 mg·L^−1^.
• A model equation for ultrasonic degassing was developed and matched to the results.

## 1 Introduction

When water is irradiated with ultrasonic waves, fine bubbles generated from the bubble nucleus repeatedly expand, contract, and grow through rectified diffusion [1], [2]. When the fine bubbles grow to a certain size, they will collapse. The generation, growth, and collapse of bubbles due to ultrasonic irradiation is called acoustic cavitation, and leads to chemical [3] and physical [4] effects. Chemical effects of ultrasonic irradiation have been reported, including degradation [5] and synthesis [6]. Ultrasonic cleaning [7] and emulsification [8] have also been reported as physical effects of ultrasonic irradiation.

After the bubbles collapse, some become fine bubbles again and some become large bubbles by coagulation or coalescence. Then, they rise to the surface of the water and disappear. While the bubbles grow due to ultrasonic irradiation, the dissolved gas in the water moves into the bubbles as gas, and the dissolved gas in the water decreases. The phenomenon of dissolved gas reduction due to ultrasonic irradiation is called ultrasonic degassing. Therefore, in addition to chemical and physical effects, acoustic cavitation can cause degassing. For ultrasonic degassing, Gondrexon et al. [9] investigated the change in dissolved oxygen concentration in water through ultrasonic irradiation of air-saturated water at a frequency of 500 kHz, a sample volume of 200–600 mL, and an applied power of 0–100 W to the transducer. They reported that the dissolved oxygen concentration decreased exponentially to a certain dissolved oxygen concentration as the ultrasonic irradiation time increased. Furthermore, Liu et al. [10] reported that hydrogen removal by degassing is effectively achieved by irradiating AZ91 magnesium alloy with ultrasonic waves at a frequency of 20 kHz during casting. Degassing is used in various fields such as oil pumps, paints, photosensitive materials, ultrasonic cleaning water, preventing microbial growth in liquid foods, and pretreatment for liquid chromatography (HPLC). Ultrasonic degassing has the advantage of reducing gases in solution shortly in a non-contact manner. However, in order to operate ultrasonic degassing under optimal conditions, it is essential to understand the effects of ultrasonic frequency and ultrasonic intensity on ultrasonic degassing. There are several reports on ultrasonic degassing but few reports on the dependence of ultrasonic degassing on the frequency and ultrasonic intensity.

In this study, the dependence of ultrasonic degassing on ultrasonic power and frequency was investigated. Ultrasonic degassing was evaluated by the dissolved oxygen concentration in the water. Ultrasonic power was defined as the energy input to the water per unit time and measured by calorimetry. The use of ultrasonic power has the advantage that common knowledge can be obtained for various ultrasonic devices with different shapes and sizes of transducers and vessels. In addition, the relationship between the concentration of dissolved oxygen and the chemical effects of ultrasonic irradiation was investigated using the potassium iodide (KI) method. Finally, a model equation for the time variation of ultrasonic degassing was developed, and the mechanism of degassing was discussed.

## 2 Experimental

### 2.1 Apparatus

**Fig. 1:**
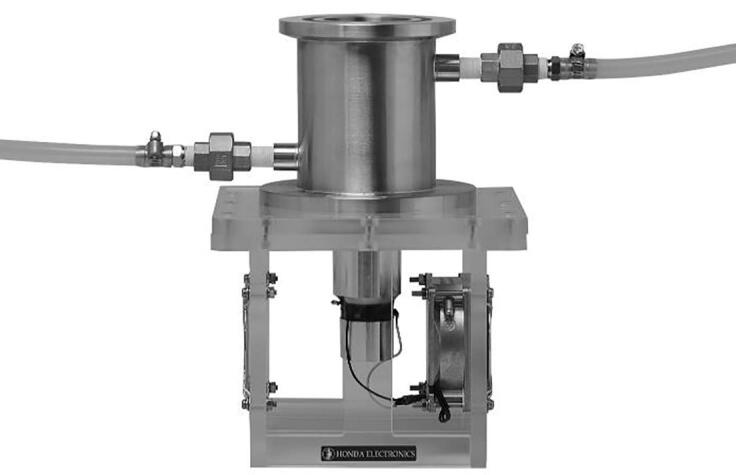
Photograph of the experimental vessel.

The vessel used for the experiments had a double-cylinder structure, and water at 298 K was circulated in the outer space of the vessel to keep the temperature of the sample in the inner space constant. Fig. 1 shows an image of the vessel. A vibration plate with a transducer was attached to the bottom of the vessel. The vessel and the vibration plate were made of SUS304 stainless steel, and the inner diameter of the vessel was 56 mm. The transducer used was a 45 mm diameter Langevin type transducer (Honda Electronics) with multi-frequencies of 22, 43, 97, and 129 kHz, and a 50 mm diameter disc-type transducer (Honda Electronics) with frequencies of 209, 305, 400, 514, 1018, and 1960 kHz. Continuous sine waves generated by a signal generator (WF1942, NF) and amplified by a power amplifier (1040L, E&I) were applied to the transducers. Except for the 305, 400, and 514 kHz transducers, a matching circuit (Honda Electronics) was inserted between the power amplifier and the transducer to match the impedance.

The effective electric power to drive the transducer was calculated from the voltage at both ends of the transducer and the current flowing through the transducer using an oscilloscope (TDS3014B, Tektronix) and a current probe (TCP202, Tektronix). A personal computer was connected to the oscilloscope and signal generator via a General Purpose Interface Bus to control the output voltage of the signal generator. In this system, the effective electric power to drive the transducer can be kept constant.

### 2.2 Measurements

#### 2.2.1 Ultrasonic power

The ultrasonic power, which is the energy input to the sample per unit time, was determined by calorimetry. The ultrasonic power *P*_U_ was calculated using the following equation:

(1) $$P_{U}=\frac{\Delta T}{\Delta t}C_{p}M,$$

where Δ*T* / Δ*t*, *C*_p_, and *M* represent the rate of temperature rise, specific heat capacity, and mass of the sample, respectively. The sample was air-saturated ultrapure water (Milli-Q Reference & Elix Essential UV5, Merck), with a volume of 100 mL, and a temperature of 298 ± 0.1 K. The temperature of the sample in the vessel was measured using a thermocouple (Type T, Takahashi Thermo) or a platinum resistor (pt100, Netsushin), and a thermometer (NR500, KEYENCE).

#### 2.2.2 Ultrasonic degassing

Ultrasonic degassing reduces the dissolved air concentration. In this study, dissolved air concentration was evaluated by dissolved oxygen concentration. The dissolved oxygen concentration of the sample before and after ultrasonic irradiation was measured using a dissolved oxygen meter (HQ-40d, HACH). Ultrapure water with a volume of 100 mL was used as the sample. The water temperature before ultrasonic irradiation was 298 ± 0.1 K. The water temperatures were 299 ± 1 K and 303 ± 5 K at 5 and 30 min of ultrasonic irradiation, respectively. The dissolved oxygen concentration of air-saturated water is affected by atmospheric pressure and water temperature. Therefore, the dissolved oxygen concentration *C* [mg·L^−1^] obtained from the dissolved oxygen meter was corrected to the dissolved oxygen concentration *C*_101.3kPa_ [mg·L^−1^] at 101.325 kPa and 298.15 K using the following equation [11], [12], [13].

(2) $$C_{101.3kPa}=\frac{C}{\frac{P}{101.325}\times \left[\frac{\left(1-\frac{P_{\mathrm{wv}}}{P}\right)\left(1-B\frac{P}{101.325}\right)}{\left(1-\frac{P_{\mathrm{wv}}}{101.325}\right)\left(1-B\right)}\right]},$$

where *P* [kPa] is the atmospheric pressure during the experiment, *P*_wv_ [kPa] is the vapour pressure, and *B* [−] is the negative value of the second pressure coefficient of virial expansion as shown in the following equation:

(3) $$P_{\mathrm{wv}}=101.325\times e^{\left(11.8571-\frac{3840.70}{T}-\frac{216961}{T^{2}}\right)},$$

(4) $$B=0.000975-1.426\times 10^{-5}\times \left(T-273.15\right)+6.436\times 10^{-8}\times {\left(T-273.15\right)}^{2},$$

where *T* [K] is the absolute temperature. If the saturated dissolved oxygen concentration is C*, the degassing rate *D* [%] can be defined as follows:

(5) $$D=\frac{\left(C^{*}-C_{101.3kPa}\right)}{C^{*}}\times 100.$$

#### 2.2.3 KI oxidation

When an aqueous solution of KI is irradiated with ultrasound, I_3_^−^ is produced by OH radicals generated by water pyrolysis. Therefore, the chemical effects of ultrasonic irradiation were evaluated using the KI method [15], which can be quantitatively evaluated by the amount of I_3_^−^ produced. The KI solution was prepared using KI (Fujifilm Wako Pure Chemicals) and ultrapure water to a concentration of 0.1 mol·L^−1^. The sample volume was 100 mL, and the sample temperature before ultrasonic irradiation was 298 ± 0.1 K. The concentration of I_3_^−^ was measured at 352 nm using a UV–Vis spectrophotometer (UV-1850, Shimadzu Corporation). The reaction rate of I_3_^−^, *k*_I3_ [mol·s^−1^], was calculated using the following equation:

(6) $$k_{I3}=\frac{\mathrm{AV}}{\varepsilon lt},$$

where *A* is the absorbance of I_3_^−^ [-], *V* is the volume of the solution [L], *ε* is the molar absorption coefficient of I_3_^−^ [L·mol^−1^·cm^−1^], *l* is the length of the cuvette [cm], and *t* is the ultrasonic irradiation time [s]. In the experiments of this study, *V*, *ε*, *l,* and *t* are 0.1 L, 26,303 L·mol^−1^·cm^−1^, 1 cm, and 30 s, respectively.

## 3 Results and discussion

### 3.1 Ultrasonic degassing

#### 3.1.1 Dependence of degassing on ultrasonic frequency

**Fig. 2:**
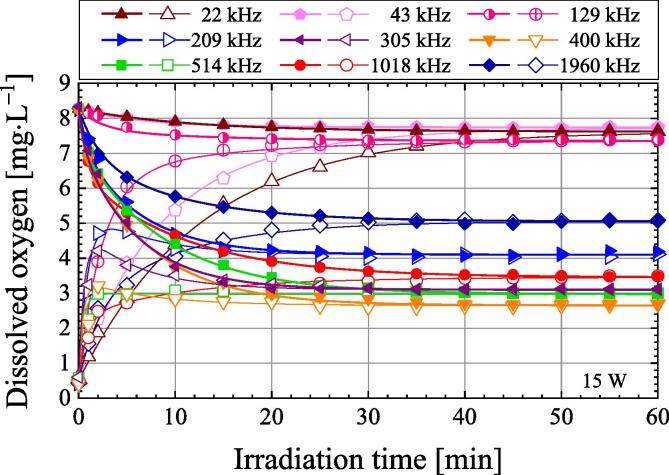
Variation of dissolved oxygen concentration as a function of irradiation time during ultrasonic irradiation of air-saturated water (closed symbols) and degassed water (open symbols) at an ultrasonic power of 15 W in the frequency range of 22–1960 kHz under 101.3 kPa.

Fig. 2 shows the change in dissolved oxygen concentration in air-saturated water and degassed water as a function of ultrasonic irradiation time. The dissolved oxygen concentration in the degassed water is 0.5 mg·L^−1^. The ultrasonic power is 15 W, the frequency ranges from 22 to 1960 kHz, and the pressure is 101.3 kPa. For air-saturated water, the dissolved oxygen concentration decreases with the ultrasonic irradiation time. After about 40 min of ultrasonic irradiation, the dissolved oxygen concentration becomes almost constant. On the other hand, in the case of degassed water, the dissolved oxygen concentration increases monotonically with the ultrasonic irradiation time, except when the frequency is 209, 305, and 400 kHz. However, for frequencies of 209, 305, and 400 kHz, the dissolved oxygen concentration increases with ultrasonic irradiation and decreases after showing the maximum dissolved oxygen concentration. The reasons for the maximum dissolved oxygen concentration are attributed to the following. At higher frequencies, such as 209, 305, and 400 kHz, the chemical reaction field has been reported to be near the water surface [14]. Furthermore, Koda et al. investigated the frequency dependence of the sonochemical reaction rate using the KI method and reported that the sonochemical efficiency was high in the frequency range of 200–500 kHz [15]. Judy et al. reported that when water was irradiated with ultrasound at a frequency of 448 kHz, the bubbles near the centre of the water in the vessel moved to near the water surface with irradiation time [16]. Immediately after ultrasonic irradiation, since the dissolved oxygen concentration near the water surface is low, which is same as the average dissolved oxygen concentration of the entire water, a lot of air dissolves in the water at the water surface. Thereafter, since many bubbles move near the water surface with ultrasonic irradiation, the dissolved oxygen concentration near the water surface becomes higher than the average dissolved oxygen concentration of the entire water. The increase rate in dissolved oxygen concentration becomes slower because dissolution amount of air into water surface decreases. Therefore, for the degassed water at 209, 305, and 400 kHz, the dissolved oxygen concentration shows a maximum value and then gradually decreases. The increase in dissolved oxygen concentration immediately after ultrasound irradiation may be related to other factors as well. The authors will investigate these factors in future studies. When air-saturated water and degassed water are irradiated with ultrasound for 40 min, the dissolved oxygen concentration remains almost the same at all frequencies. Therefore, the dissolved oxygen concentration after a long period of ultrasonic irradiation, such as 60 min, depends on the frequency, not the initial dissolved oxygen concentration.

**Fig. 3:**
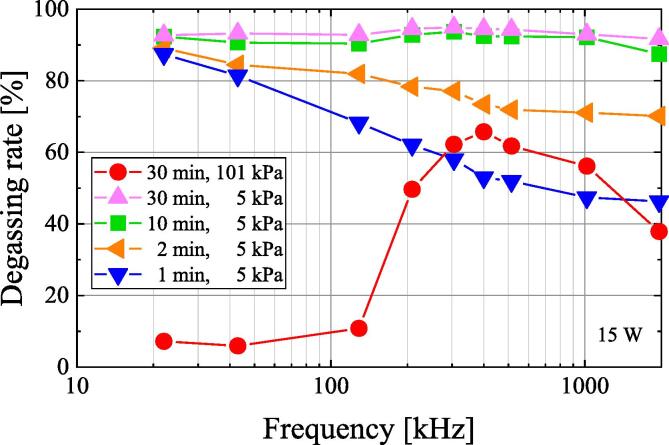
Frequency dependence of the degassing rate of air-saturated water after 1, 2, 10, and 30 min of ultrasonic irradiation at an ultrasonic power of 15 W under 101 kPa and 5 kPa.

Fig. 3 shows the frequency dependence of the degassing rate. The ultrasonic irradiation time, ultrasonic power, and pressure were set to 30 min, 15 W, and 101.3 kPa, respectively. The degassing rate can be calculated from the dissolved oxygen concentration using Eq. (5). Therefore, the rate of ultrasonic degassing is high in the frequency range of 200 kHz to 1 MHz. When water is irradiated with ultrasonic waves, fine bubbles generated from the bubble nucleus repeatedly expand, contract, and grow through rectified diffusion [1], [2]. When the fine bubbles grow to a certain size, they collapse. The generation, growth, and collapse of bubbles due to ultrasonic irradiation is called cavitation. Because the collapse of the fine bubbles is caused by semiadiabatic compression, the field inside the fine bubbles reaches a high temperature and high pressure. This generates various radical species in and near the fine bubbles, which produce chemical effects [3]. Furthermore, while the bubbles are growing due to ultrasonic irradiation, the dissolved gas in the water moves into the bubbles as gas and the dissolved gas in the water decreases. Ultrasonic degassing occurs because of gas transfer between the bubble surface and the water surface to achieve equilibrium [9]. Therefore, sonochemical efficiency and ultrasonic degassing depend on the number of cavitation bubbles, because chemical reactions and degassing occur by cavitation. This indicated that the frequency dependence of ultrasonic degassing may be similar to that of sonochemical efficiency.

#### 3.1.2 Dependence of degassing on ultrasonic frequency under 5 kPa

**Fig. 4:**
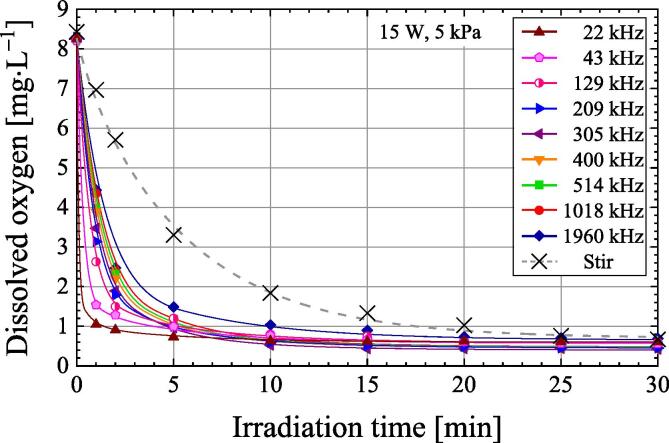
Variation of dissolved oxygen concentration as a function of irradiation time during the ultrasonic irradiation of air-saturated water at an ultrasonic power of 15 W in the frequency range of 22–1960 kHz and variation of dissolved oxygen concentration by stirring under 5 kPa.

Fig. 4 shows the change in dissolved oxygen concentration as a function of ultrasonic irradiation time under a reduced pressure of 5 kPa. Air-saturated water is irradiated at frequencies of 22–1960 kHz and ultrasonic power of 15 W. Ultrasonic irradiation under reduced pressure of 5 kPa results in a rapid and significant decrease in dissolved oxygen concentration compared to 101.3 kPa. Fig. 4 also shows the dissolved oxygen concentration when air-saturated water is stirred under a reduced pressure of 5 kPa. Stirring was performed with a 30 mm long magnetic stirrer at a rotational speed of about 400 rpm. Again, under a reduced pressure of 5 kPa, the dissolved oxygen concentration decreases more quickly with ultrasonic irradiation than when it is stirred at other frequencies.

Fig. 3 shows the frequency dependence of the degassing rate under reduced pressure of 5 kPa. The degassing rate of air-saturated water was calculated using Eq. (5) after 1, 2, 10, and 30 min of ultrasonic irradiation at ultrasonic power of 15 W under reduced pressure of 5 kPa. The degassing rate by ultrasonic irradiation for more than 10 min is almost the same for all frequencies. When air-saturated water is irradiated with ultrasonic waves for 1 to 2 min at a reduced pressure of 5 kPa, the degassing rate increases with decreasing frequency. The frequency dependence of the degassing rate at 5 kPa is different from that of 101.3 kPa. When the cavitation bubble collapses, a jet stream is generated near the bubble. When the jet stream stirs the water, degassing is accelerated under reduced pressure. The jet stream accelerates with decreasing frequency [17], [18]. Therefore, the lower the frequency, the stronger the stirring by jet stream and more degassing is promoted. Additionally, when cavitation bubbles collapse, the size of the bubble increases with decreasing frequency. Furthermore, due to the primary Bjerknes force, which is a type of radiation force generated in standing waves, bubbles are trapped in the antinode of the sound pressure in standing waves [19], [20]. When two bubbles expand and contract in the same phase, they are attracted by the secondary Bjerknes force, which is a kind of radiation force [19], [20]. Thus, the bubbles aggregate and coalesce due to the primary and secondary Bjerknes forces and their size increase. The primary and secondary Bjerknes forces are larger at lower frequencies. Furthermore, under reduced pressure, the bubbles become larger in size. Therefore, at a lower frequency under a reduced pressure of 5 kPa, the degassing rate increases because the stirring by jet stream generates more bubbles, and they also grow larger.

#### 3.1.3 Dependence of degassing on ultrasonic power

**Fig. 5:**
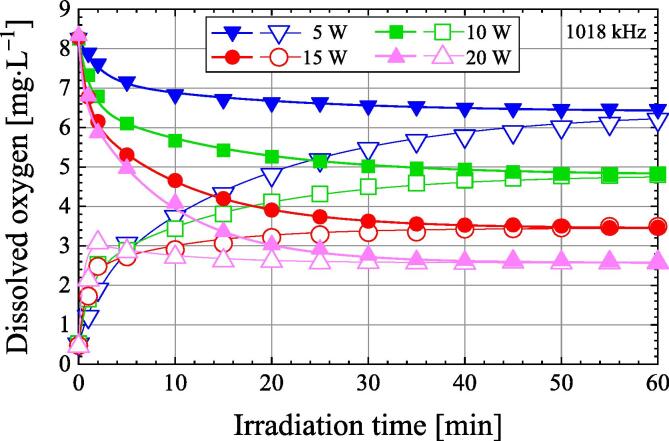
Variation of dissolved oxygen concentration as a function of irradiation time during the ultrasonic irradiation of air-saturated water (closed symbols) and degassed water (open symbols) in the ultrasonic power range of 5–20 W at a frequency of 1018 kHz under 101.3 kPa.

Fig. 5 shows the variation of dissolved oxygen concentration with ultrasonic irradiation time at 1018 kHz frequency and different ultrasonic powers. Air-saturated water and degassed water with a dissolved oxygen concentration of about 0.5 mg·L^−1^ were used as samples. The ultrasonic powers are 5, 10, 15, and 20 W. In the case of air-saturated water, the dissolved oxygen concentration decreases with ultrasonic irradiation time and becomes almost constant after about 60 min of ultrasonic irradiation. On the other hand, in the case of degassed water, the dissolved oxygen concentration increases monotonically with the ultrasonic irradiation time, except when the ultrasonic power is 20 W. However, at the ultrasonic power of 20 W, the dissolved oxygen concentration increases with the ultrasonic irradiation time, reaches a maximum value, and then decreases. Probably this is the same reason as in the case of ultrasonic irradiation of degassed water at 209, 305, and 400 kHz in Fig. 2. We will investigate the reason for this in future studies. When degassed water is irradiated with ultrasonic waves at high ultrasonic power, the dissolved oxygen concentration does not increase monotonically with the ultrasonic irradiation time. After 60 min of ultrasonic irradiation, the dissolved oxygen concentrations of air-saturated water and degassed water are almost the same, except for 5 W. Presumably, if air-saturated water and degassed water are irradiated with 5 W ultrasonic power for a long time, the dissolved oxygen concentration in both will be the same. Thus, after prolonged ultrasonic irradiation, the dissolved oxygen concentration depends on the ultrasonic power and not the initial dissolved oxygen.

**Fig. 6:**
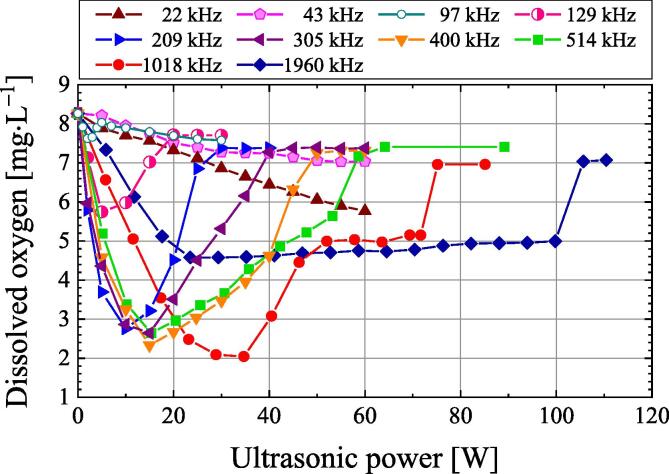
Dependence of the dissolved oxygen concentration on the ultrasonic power when air-saturated water is irradiated with ultrasound at frequencies at 22–1960 kHz for 30 min.

Fig. 6 shows the dependence of the dissolved oxygen concentration on the ultrasonic power when air-saturated water is irradiated with ultrasound at frequencies at 22–1960 kHz for 30 min. At frequencies of 22 and 43 kHz, the dissolved oxygen concentration decreases monotonically with increasing ultrasonic power. The dissolved oxygen concentration decreases to a minimum value at other frequencies as the ultrasonic power increases from zero. For example, when the frequency is 400 kHz and 1018 kHz, the dissolved oxygen concentration shows minimum values of 2.3 and 2.0 mg·L^−1^, respectively. After that, as the ultrasonic power increases, the dissolved oxygen concentration increases to 7.0 mg·L^−1^ or higher. The reason why the dissolved oxygen concentration becomes higher above the ultrasonic power at which the dissolved oxygen concentration is minimal is that the water surface area increases due to the significant movement of the water surface caused by the increased radiation force by ultrasound, and the water is agitated near the water surface. Furthermore, the dissolved oxygen concentration increases rapidly when the ultrasonic power is 64.2, 75.2, and 105.7 W at frequencies 514, 1018, and 1960 kHz, respectively. As the ultrasonic intensity increases, the chemical reaction rate increases due to an increase in the number of cavitation bubbles and an increase in the temperature within the cavitation bubbles [15], [21], [22]. However, it was reported that when the ultrasonic intensity was quite high, the chemical reaction rate was greatly reduced [23], [24]. The decrease in the reaction rate despite an increase in ultrasonic power is called quenching phenomenon. The authors observed the quenching phenomenon of chemical reactions by the KI method when ultrasonic powers at the frequency of 514, 1018 and 1960 kHz were 64, 75 and 106 W, respectively. These ultrasonic powers are almost same as those at the rapid increase in the dissolved oxygen concentration in Fig. 6. Therefore, the authors assumed that the rapid increase in the dissolved oxygen concentration was related to the quenching phenomenon. Due to the quenching at high ultrasonic powers, bubbles do not grow and cavitation does not occur except at 22 and 43 kHz. Therefore, the dissolved oxygen concentration increased significantly due to quenching at high ultrasonic power. When the optimum ultrasonic power is applied to air-saturated water, the minimum dissolved oxygen values are almost the same in the frequency range of 200 kHz to 1 MHz.

### 3.2 Dependence of cavitation on initial dissolved oxygen concentration

The concentration of dissolved air in water, which is reduced by ultrasonic degassing, may affect the amount of ultrasonic cavitation. Therefore, we investigated the relationship between the dissolved air concentration in water before ultrasonic irradiation and the amount of ultrasonic cavitation to determine the effect of dissolved air concentration in water on ultrasonic cavitation. The dissolved air concentration in water and the amount of ultrasonic cavitation were evaluated by the concentration of dissolved oxygen and the reaction rate of I_3_^−^ using the KI method in Eq. (6), respectively. The dissolved oxygen concentration before ultrasonic irradiation is defined as the initial dissolved oxygen concentration.

**Fig. 7:**
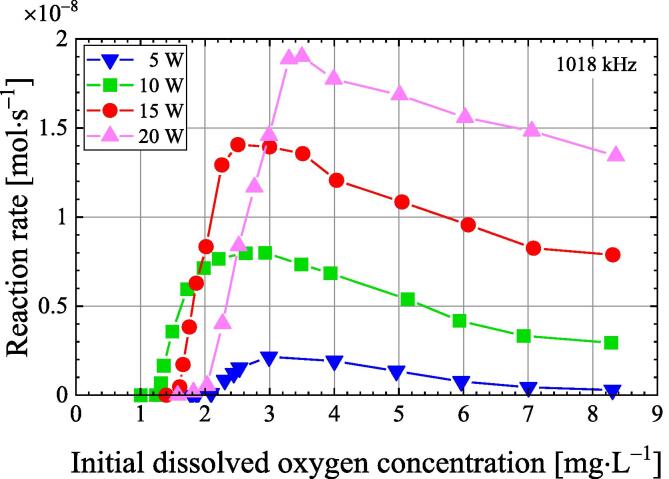
The reaction rate of I_3_^−^ vs. initial dissolved oxygen concentration at ultrasonic power of 5, 10, 15, and 20 W under 101.3 kPa.

Fig. 7 shows the relationship between the initial dissolved oxygen concentration and the reaction rate of I_3_^−^ after 30 s of ultrasonic irradiation at a frequency of 1018 kHz and ultrasonic power of 5, 10, 15, and 20 W. The reaction rates of I_3_^−^ are maximum when the initial dissolved oxygen concentrations at 5, 10, 15, and 20 W are 3.0, 2.5, 2.5, and 3.5 mg·L^−1^, respectively. Thus, the sonochemical reaction in degassed water of about 3 mg·L^−1^ is faster than in air-saturated water. When the initial dissolved oxygen concentration is as high as the air saturation, the scattering and attenuation of ultrasound by many bubbles increases and the sound pressure decreases [25]. The reaction rate decreases significantly after showing its maximum as initial dissolved oxygen concentration decreases. Furthermore, the reaction rate is almost zero below the threshold of dissolved oxygen concentration where the initial dissolved oxygen at the ultrasonic power of 5, 10, 15, and 20 W are 1.8, 1.2, 1.4, and 1.6 mg·L^−1^, respectively. Therefore, if the initial dissolved oxygen concentration is below this threshold, cavitation will not occur.

When the dissolved oxygen concentration is low, the fine bubbles generated by ultrasonic irradiation do not grow by rectified diffusion and dissolve in water [2]. In this case, the higher the sound pressure, the more the number of bubbles dissolved in the water. Therefore, the initial dissolved oxygen concentration threshold increases with increasing ultrasonic power, except for 5 W. When the ultrasonic power is 5 W, the only part of the sound field with an acoustic pressure above the cavitation threshold is near the centre of the sample in the vessel. The spatial volume of this sound field is smaller than when the ultrasonic power is 10 W or higher. This is because some bubbles generated within the spatial volume will move from the spatial volume due to ultrasonic stirring and disappear into the water without growing. Therefore, the initial dissolved oxygen concentration threshold at an ultrasonic power of 5 W is greater than the threshold at an ultrasonic power of 10 W or higher. Since cavitation will not occur if the initial dissolved oxygen concentration is less than 1.2 to 1.8 mg·L^−1^, these are the lower limits of dissolved oxygen concentration by ultrasonic degassing, as shown in Fig. 6.

### 3.3 Model of degassing

#### 3.3.1 Model

**Fig. 8:**
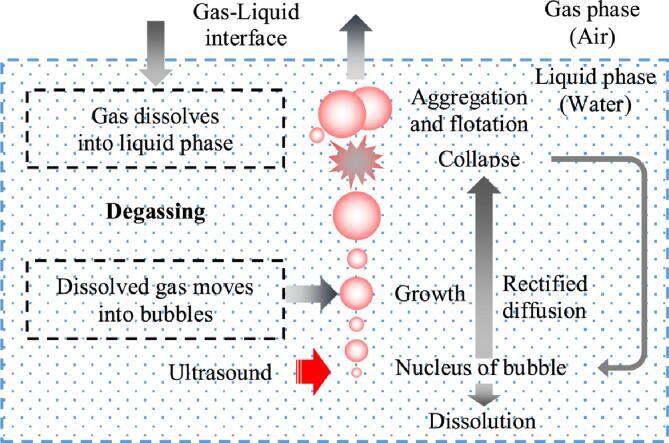
Model of degassing mechanism.

Fig. 8 shows a model of degassing by ultrasonic irradiation. When ultrasound propagates through water, fine bubbles generated from the bubble nucleus in the water grow while repeatedly contracting and expanding due to the high and low sound pressure caused by the ultrasound propagation. Once the fine bubbles have grown to a certain size, they collapse. This phenomenon of bubble generation, growth and collapse is called ‘acoustic cavitation’. Some collapsed bubbles will coalesce into larger bubbles and rise to the liquid surface, while some will become fine bubbles and cavitate again. As dissolved air moves into the bubbles during bubble growth, the concentration of dissolved air in the water decreases. On the other hand, if the dissolved air concentration is lower than the saturated dissolved air concentration, the air moves into the water above the water surface and dissolves in the water. Thus, degassing is caused by the balance between the movement of dissolved air into the bubbles due to the growth of bubbles under ultrasonic irradiation and the dissolution of air above the water surface.

A model equation for dissolved air concentration by ultrasound irradiation is introduced to evaluate the dissolved air concentration in terms of dissolved oxygen concentration. The time variation of the dissolved oxygen concentration *C* [mg·L^−1^] can be expressed as follows:

(7) $$\begin{array}{c} \frac{\mathrm{dC}}{\mathrm{dt}}=K_{L}a\left(C^{*}-C\right)-B_{1}\left(C-C_{TH}\right)+be^{-B_{2}t},\\ \;\left(when\;C<C_{TH},B_{1}=0\right), \end{array}$$

where *t* [s] is the time, the first term on the right-hand side of Eq. (7) is the change in dissolved oxygen concentration due to the transfer of oxygen from the gas phase to the liquid phase on the water surface, C* [mg·L^−1^] is the saturated dissolved oxygen concentration, and *K*_L_ [m·s^−1^] and *a* [m^2^·m^−3^] are the mass transfer coefficient and specific surface area, respectively. The second term on the right-hand side is the change in dissolved oxygen concentration due to the bubbles generated by ultrasonic irradiation through rectified diffusion. *C*_TH_ [mg·L^−1^] is the dissolved oxygen concentration threshold for bubble growth or dissolution by rectified diffusion. *B*_1_ [s^−1^] is the degassing volumetric mass transfer coefficient for bubble growth. The third term on the right-hand side enhances the time variation of dissolved oxygen concentration due to ultrasound immediately after ultrasound irradiation, *b* [mg·L^−1^·s^−1^] is the magnitude of the third term, and *B*_2_ [s^−1^] is the volumetric mass transfer coefficient of the third term. When *C*_TH_ = 0, the first and second terms on the right side of Eq. (7) are the same as an equation reported by Gondrexon et al. [9]. Thus, the solution to Eq. (7) can be expressed as the sum of two exponential functions as follows:

(8) $$\begin{array}{c} C=\left(C_{0}-\frac{K_{L}aC^{*}+B_{1}C_{TH}}{K_{L}a+B_{1}}-\frac{b}{K_{L}a+B_{1}-B_{2}}\right)e^{-\left(K_{L}a+B_{1}\right)t}+\frac{K_{L}aC^{*}+B_{1}C_{TH}}{K_{L}a+B_{1}}\\ \;+\frac{b}{K_{L}a+B_{1}-B_{2}}e^{-B_{2}t},\;\left(when\;C<C_{\mathrm{TH}},B_{1}=0\right), \end{array}$$

where *C*_0_ is the initial dissolved oxygen concentration. If the dissolved oxygen concentration at infinite time *t* is *C*_∞_, then *C*_∞_, and Eq. (8) can be expressed as follows:

(9) $$C_{\infty }=\frac{K_{L}aC^{*}+B_{1}C_{TH}}{K_{L}a+B_{1}},$$

(10) $$\begin{array}{c} C=\left(C_{0}-C_{\infty }-\frac{b}{K_{L}a+B_{1}-B_{2}}\right)e^{-\left(K_{L}a+B_{1}\right)t}+C_{\infty }+\frac{b}{K_{L}a+B_{1}-B_{2}}e^{-B_{2}t},\\ \;(when\;C<C_{\mathrm{TH}},B_{1}=0). \end{array}$$

#### 3.3.2 Model results

The calculation results obtained from Eq. (10) are plotted as solid lines in Fig. 2, Fig. 4, Fig. 5. By adding the third term to the right-hand side of Eq. (7), the calculation results obtained from Eq. (10) are in good agreement with the experimental data. However, the physical meaning of the third term on the right-hand side of Eq. (7) is not yet known.

**Fig. 9:**
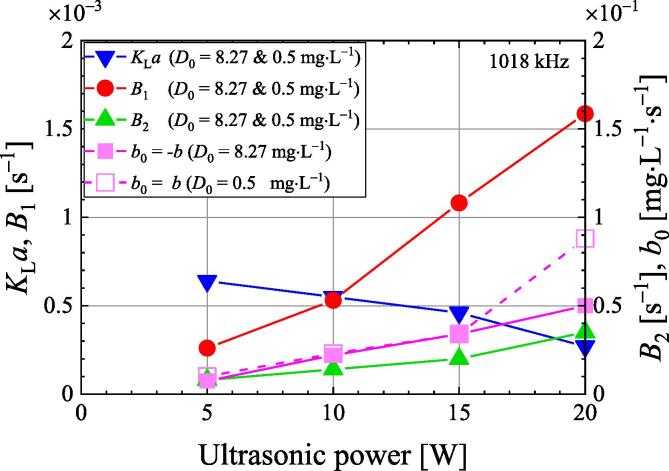
Dependence of various parameters of the degassing model on ultrasonic power at a frequency of 1018 kHz under 101.3 kPa.

Fig. 9 shows the ultrasonic power dependence of each parameter in Eq. (10). Experimental data in Fig. 5 were used to obtain these parameters. The volumetric mass transfer coefficient *B*_1_ on the bubble surface is independent of the initial dissolved oxygen concentration. It becomes larger with increasing ultrasonic power because the number of cavitation bubbles increases with increasing ultrasonic power. The volumetric mass transfer coefficient *K*_L_*a* on the liquid surface is also independent of the initial dissolved oxygen concentration and decreases with increasing ultrasonic power. The higher the ultrasonic power, the higher the dissolved oxygen concentration near the water surface due to enhanced degassing. As a result, mass transfer on the water surface decreases. The volumetric mass transfer coefficient *B*_2_ is also independent of the initial dissolved oxygen concentration and increases with increasing ultrasonic power. When the initial dissolved oxygen concentration is saturated, the enhancement coefficient *b* is negative, when the initial dissolved oxygen concentration is 0.5 mg·L^−1^, *b* is positive. The absolute value of *b* is almost independent of the initial dissolved oxygen concentration except for 20 W and increases with increasing ultrasonic power. When the ultrasonic power is 20 W, the absolute value of *b* is larger at the initial dissolved oxygen concentration of 0.5 mg·L^−1^ than at the initial dissolved oxygen concentration of air saturation. By increasing the ultrasonic power, degassing increases immediately after ultrasound irradiation. Focusing on the volumetric mass transfer coefficient *K*_L_*a* and *B*_1_, the ultrasonic power dependence of degassing speed is most likely due to the number of cavitation bubbles.

**Fig. 10:**
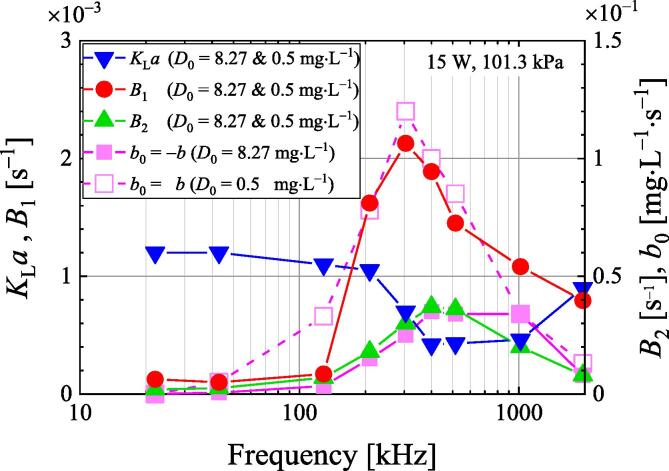
Ultrasonic frequency dependence of various parameters of the degassing model at an ultrasonic power of 15 W under 101.3 kPa.

Fig. 10 shows the frequency dependence of each parameter in Eq. (10), and we used experimental data in Fig. 2 to obtain these parameters. The volumetric mass transfer coefficient *K*_L_*a*, *B*_1_, and *B*_2_ are independent of the initial dissolved oxygen concentration. The volumetric mass transfer coefficient *B*_1_ is very small below 130 kHz, reaches a maximum at 300 kHz, and decreases with increasing frequencies above 300 kHz. The volumetric mass transfer coefficient *K*_L_*a* tends to decrease with increasing frequency, especially with the *K*_L_*a* value and decreases in the frequency range of 300 kHz to 1 MHz. This is probably the same reason as the relationship between *K*_L_*a* and *B*_1_ in Fig. 9.

The frequency dependence of the volumetric mass transfer coefficient *B*_2_ is a characteristic that shows a maximum value at 400 kHz. The frequency dependence of the absolute value of the enhancement coefficient *b* has a characteristic that shows maximum values at 300 or 400 kHz. The coefficient *b* in degassed water with 0.5 mg·L^−1^ indicates a value greater than the air saturation condition. The frequency dependence of the volumetric mass transfer coefficients *B*_1_ and *B*_2_, and enhancement coefficient *b* shows a similar trend to that of the degassing rate under 101 kPa in Fig. 3, suggesting that degassing is dominated by *B*_1_, *B*_2_, and *b* and may depend on the number and growth of bubbles.

**Fig. 11:**
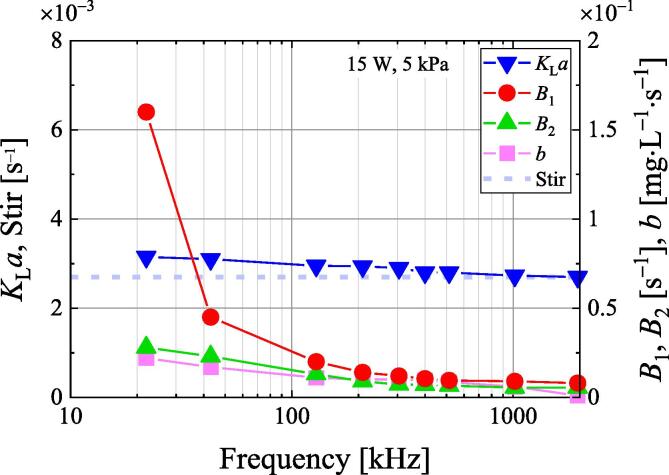
Ultrasonic frequency dependence of various parameters of the degassing model at an ultrasonic power of 15 W under 5 kPa.

Fig. 11 shows the various parameters of Eq. (10) when using the frequency dependence of the time variation of the dissolved oxygen concentration under pressure below 5 kPa in Fig. 4. At lower frequencies, the volumetric mass transfer coefficient *B*_1_ becomes very large, while the volumetric mass transfer coefficients *K*_L_*a* and *B*_2_, and enhancement coefficient *b* slightly increase. Since the volumetric mass transfer coefficient, *K*_L_*a* is slightly larger than that during stirring, the effect of mass transfer on the water surface is considered small. However, the authors believe that lowering the frequency under reduced pressure will increase the size of the bubbles and increase the degassing rate. By using the degassing model, the degassing mechanism can be better understood. In the future, we will investigate the physical meaning of the third term on the right-hand side of Eq. (7) so that we can more easily express the volumetric mass transfer coefficient *B*_2_ and the enhancement coefficient *b*.

## 4 Conclusions

The time dependence of dissolved oxygen concentration on ultrasonic irradiation was investigated in air-saturated water and degassed water with a sample volume of 100 mL. The dissolved oxygen concentration varied with ultrasonic irradiation time and became constant after about 60 min. The dissolved oxygen concentration, which became constant as the irradiation time increased, depended on the frequency and ultrasonic power. However, it did not depend on the initial dissolved oxygen concentration. Ultrasonic irradiation under reduced pressure enhanced degassing, and under 101.3 kPa, the degassing rate was high in the frequency range of 200 kHz to 1 MHz. This frequency dependence was similar to the sonochemical efficiency obtained by the KI method. However, under a reduced pressure of 5 kPa, the degassing rate was higher at lower frequencies.

The dependence of dissolved oxygen concentration on ultrasonic power was investigated by irradiating air-saturated water with ultrasound in the frequency range of 22 to 1960 kHz. Except for frequencies 22 and 43 kHz, the ultrasonic powers maximised the degassing rate, and the degassing rate decreased above these ultrasonic powers. Thus, it was found that ultrasonic degassing was highly dependent on ultrasonic power.

The sonochemical reaction rate was investigated by the KI method at different dissolved air concentrations before ultrasonic irradiation. Cavitation did not occur when the initial dissolved oxygen concentration was less than 2.2 mg·L^−1^. Therefore, the lower limit of ultrasonic degassing under 101.3 kPa was found to be 2 mg·L^−1^ dissolved oxygen concentration.

A model equation for the time variation of dissolved oxygen concentration due to ultrasonic irradiation was developed. This model equation predicted the experimental results by adding a term that enhanced the time variation of dissolved oxygen concentration immediately after ultrasound irradiation.

### CRediT authorship contribution statement

**Yoshiyuki Asakura:** Conceptualization, Methodology, Software, Validation, Formal analysis, Investigation, Resources, Data curation, Writing – original draft, Writing – review & editing, Visualization, Supervision, Project administration. **Keiji Yasuda:** Validation, Resources, Writing – review & editing, Funding acquisition.

## Declaration of Competing Interest

The authors declare that they have no known competing financial interests or personal relationships that could have appeared to influence the work reported in this paper.
